# Dimethyl 1-(4-cyano­benz­yl)-1*H*-pyrazole-3,5-dicarboxyl­ate

**DOI:** 10.1107/S1600536809015578

**Published:** 2009-04-30

**Authors:** Jie Xiao, Hong Zhao

**Affiliations:** a Ordered Matter Science Research Center, College of Chemistry and Chemical, Engineering, Southeast University, Nanjing 210096, People’s Republic of China

## Abstract

The title compound, C_15_H_13_N_3_O_4_, was synthesized from dimethyl 1*H*-pyrazole-3,5-dicarboxyl­ate and 4-(bromo­meth­yl)benzonitrile. The inter­planar angle between the pyrazole and cyano­benzyl ring planes is 71.74 (17)° and an intramolecular C—H⋯O interaction occurs. The crystal structure is stabilized by π–π stacking inter­actions between the neighbouring pyrazole and benzene rings [centroid–centroid distances of 3.5074 (16) and 3.9401 (15) Å, respectively]. One of the methyl groups is disordered over two positions of equal occupancy.

## Related literature

For the biological activity of pyrazoles, see: Chambers *et al.* (1985 ▶); Lee *et al.* (1989 ▶). Nitrile derivatives are important materials in the synthesis of some heterocyclic mol­ecules (Radl *et al*., 2000 ▶). For related structures, see: Dai *et al.* (2008 ▶); Fu & Zhao (2007 ▶); Xiao & Zhao (2008*a* ▶,*b* ▶,*c* ▶).
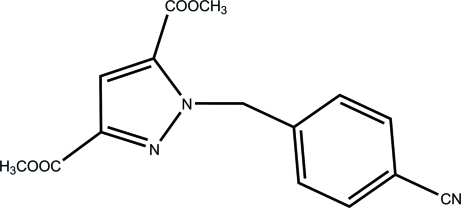


## Experimental

### 

#### Crystal data

C_15_H_13_N_3_O_4_
*M* *_r_* = 299.28
Triclinic, 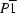

*a* = 7.4981 (13) Å
*b* = 9.1753 (9) Å
*c* = 12.2884 (18) Å
α = 69.820 (5)°
β = 88.900 (6)°
γ = 68.818 (5)°
*V* = 734.51 (18) Å^3^
*Z* = 2
Mo *K*α radiation
μ = 0.10 mm^−1^
*T* = 292 K
0.35 × 0.30 × 0.25 mm

#### Data collection

Rigaku SCXmini diffractometer
Absorption correction: multi-scan (*CrystalClear*; Rigaku, 2005 ▶) *T*_min_ = 0.968, *T*_max_ = 0.975
7543 measured reflections
3330 independent reflections
1972 reflections with *I* > 2σ(*I*)
*R*_int_ = 0.038

#### Refinement

*R*[*F* ^2^ > 2σ(*F* ^2^)] = 0.060
*wR*(*F* ^2^) = 0.161
*S* = 1.03
3330 reflections
202 parameters
H-atom parameters constrained
Δρ_max_ = 0.26 e Å^−3^
Δρ_min_ = −0.15 e Å^−3^

### 

Data collection: *CrystalClear* (Rigaku, 2005 ▶); cell refinement: *CrystalClear*; data reduction: *CrystalClear*; program(s) used to solve structure: *SHELXS97* (Sheldrick, 2008 ▶); program(s) used to refine structure: *SHELXL97* (Sheldrick, 2008 ▶); molecular graphics: *SHELXTL* (Sheldrick, 2008 ▶); software used to prepare material for publication: *SHELXTL*.

## Supplementary Material

Crystal structure: contains datablocks I, global. DOI: 10.1107/S1600536809015578/fb2152sup1.cif

Structure factors: contains datablocks I. DOI: 10.1107/S1600536809015578/fb2152Isup2.hkl

Additional supplementary materials:  crystallographic information; 3D view; checkCIF report

## Figures and Tables

**Table 1:** Hydrogen-bond geometry (Å, °)

*D*—H⋯*A*	*D*—H	H⋯*A*	*D*⋯*A*	*D*—H⋯*A*
C6—H6*A*⋯O2	0.97	2.38	2.966 (3)	119
C14—H14*A*⋯O4^i^	0.96	2.41	3.312 (4)	156

Symmetry code: (i) 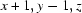.

**Fig. 1.**
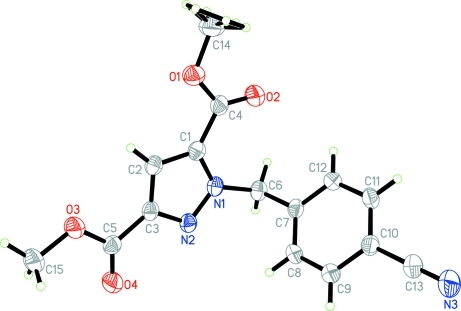
The structure of the title molecule, showing the atomic numbering scheme. The displacement ellipsoids are drawn at the 30% probability level.

## supplementary crystallographic information

### Comment

Pyrazole-related molecules have attracted considerable attention due to their biological activities (Lee *et al.*, 1989; Chambers *et al.*, 1985). In addition, the nitrile derivatives are important materials in the synthesis of some heterocyclic molecules (Radl *et al.*, 2000). Recently, we have reported a few benzonitrile compounds (Dai *et al.*,2008; Fu *et al.*, 2007; Xiao *et al.*,2008a, 2008b 2008c). As an extension of our work on the structural characterization of the nitrile compounds, the structure of the title compound is reported here. The bond lengths and angles have normal values. The interplanar angle between the pyrazole and cyanobenzyloxy ring planes is 71.74 (17) °. The crystal structure is stabilized by week interactions: C—H···O interactions, C—H···π-electron ring interactions (Tab. 1) and π-π-electron ring stacking interactions (Tab. 2).

### Experimental

1*H*-pyrazole-3,5-dicarboxylic acid dimethyl ester (0.185 mg, 1 mmol) and 4-(bromomethyl)benzonitrile (0.196 mg, 1 mmol) were dissolved in acetone (10 ml) in the presence of K_2_CO_3_ (0.138 mg, 1 mmol) and heated under reflux for 1 day. After the mixture had been cooled to room temperature, the solution was filtered and the solvent removed in vacuum to afford a white precipitate of the title compound. Colourless prisms (average size: 0.8×1.2×1.0 mm) suitable for X-ray diffraction were obtained by slow evaporation in 7 days from a solution of 100 mg of the crude product in 15 ml of diethylether.

### Refinement

All the hydrogens were discernible in the difference electron density maps. In the case of the methyl C14 the corresponding triplet of maxima was broad with shallow saddles between them. All the hydrogens were situated into the idealized positions and those of C14 were modelled as disordered with two triplets of the hydrogens with equal occupation rotated by 60° to each other. The hydrogens were treated in the riding mode approximation: C_aryl_-H, C_methylene_-H, C_methyl_-H = 0.93, 097 and 0.96 Å, respectively. U_iso_(H_aryl_)=1.2U_eq_(C_aryl_); U_iso_(H_methylene_)=1.2U_eq_(C_methylene_); U_iso_(H_methyl_)=1.5U_eq_(C_methyl_).

### Crystal data

**Table:**

C_15_H_13_N_3_O_4_	*Z* = 2
*M**_r_* = 299.28	*F*(000) = 312
Triclinic, *P*1	*D*_x_ = 1.353 Mg m^−^^3^
Hall symbol: -P 1	Mo *K*α radiation, λ = 0.71073 Å
*a* = 7.4981 (13) Å	Cell parameters from 1468 reflections
*b* = 9.1753 (9) Å	θ = 2.6–27.4°
*c* = 12.2884 (18) Å	µ = 0.10 mm^−^^1^
α = 69.820 (5)°	*T* = 292 K
β = 88.900 (6)°	Prism, colourless
γ = 68.818 (5)°	0.35 × 0.30 × 0.25 mm
*V* = 734.51 (18) Å^3^	

### Data collection

**Table:**

Rigaku SCXmini diffractometer	3330 independent reflections
Radiation source: fine-focus sealed tube	1972 reflections with *I* > 2σ(*I*)
graphite	*R*_int_ = 0.038
Detector resolution: 13.6612 pixels mm^-1^	θ_max_ = 27.4°, θ_min_ = 2.9°
ω scans	*h* = −9→9
Absorption correction: multi-scan (*CrystalClear*; Rigaku, 2005)	*k* = −11→11
*T*_min_ = 0.968, *T*_max_ = 0.975	*l* = −15→15
7543 measured reflections	

### Refinement

**Table:**

Refinement on *F*^2^	Secondary atom site location: difference Fourier map
Least-squares matrix: full	Hydrogen site location: difference Fourier map
*R*[*F*^2^ > 2σ(*F*^2^)] = 0.060	H-atom parameters constrained
*wR*(*F*^2^) = 0.161	*w* = 1/[σ^2^(*F*_o_^2^) + (0.0703*P*)^2^ + 0.0489*P*] where *P* = (*F*_o_^2^ + 2*F*_c_^2^)/3
*S* = 1.03	(Δ/σ)_max_ < 0.001
3330 reflections	Δρ_max_ = 0.26 e Å^−^^3^
202 parameters	Δρ_min_ = −0.15 e Å^−^^3^
0 restraints	Extinction correction: *SHELXL97* (Sheldrick, 2008), Fc^*^=kFc[1+0.001xFc^2^λ^3^/sin(2θ)]^-1/4^
62 constraints	Extinction coefficient: 0.023 (5)
Primary atom site location: structure-invariant direct methods	

### Special details

**Table:**

Geometry. All e.s.d.'s (except the e.s.d. in the dihedral angle between two l.s. planes) are estimated using the full covariance matrix. The cell e.s.d.'s are taken into account individually in the estimation of e.s.d.'s in distances, angles and torsion angles; correlations between e.s.d.'s in cell parameters are only used when they are defined by crystal symmetry. An approximate (isotropic) treatment of cell e.s.d.'s is used for estimating e.s.d.'s involving l.s. planes.
Refinement. Refinement of *F*^2^ against ALL reflections. The weighted *R*-factor *wR* and goodness of fit *S* are based on *F*^2^, conventional *R*-factors *R* are based on *F*, with *F* set to zero for negative *F*^2^. The threshold expression of *F*^2^ > σ(*F*^2^) is used only for calculating *R*-factors(gt) *etc*. and is not relevant to the choice of reflections for refinement. *R*-factors based on *F*^2^ are statistically about twice as large as those based on *F*, and *R*- factors based on ALL data will be even larger.

### Fractional atomic coordinates and isotropic or equivalent isotropic displacement parameters (Å^2^)

**Table:**

	*x*	*y*	*z*	*U*_iso_*/*U*_eq_	Occ. (<1)
O1	0.5650 (2)	−0.3342 (2)	0.04531 (15)	0.0655 (5)	
O2	0.5168 (3)	−0.3555 (2)	0.22929 (16)	0.0819 (6)	
O3	0.0543 (3)	0.2623 (2)	−0.24779 (14)	0.0728 (5)	
O4	−0.1136 (3)	0.4051 (2)	−0.14182 (15)	0.0793 (6)	
N1	0.2237 (2)	−0.0155 (2)	0.13109 (15)	0.0491 (5)	
N2	0.0980 (2)	0.1342 (2)	0.06034 (16)	0.0503 (5)	
N3	0.8678 (4)	0.2304 (3)	0.4352 (3)	0.0966 (9)	
C1	0.3321 (3)	−0.1079 (3)	0.06994 (19)	0.0468 (5)	
C2	0.2698 (3)	−0.0139 (3)	−0.04608 (18)	0.0479 (5)	
H2	0.3143	−0.0430	−0.1098	0.057*	
C3	0.1264 (3)	0.1341 (3)	−0.04801 (19)	0.0472 (5)	
C4	0.4791 (3)	−0.2778 (3)	0.1258 (2)	0.0530 (6)	
C5	0.0081 (3)	0.2824 (3)	−0.1475 (2)	0.0515 (6)	
C6	0.2365 (3)	−0.0489 (3)	0.25677 (19)	0.0550 (6)	
H6A	0.2761	−0.1686	0.2991	0.066*	
H6B	0.1102	0.0062	0.2765	0.066*	
C7	0.3779 (3)	0.0122 (3)	0.29447 (18)	0.0479 (5)	
C8	0.3384 (3)	0.1819 (3)	0.2585 (2)	0.0576 (6)	
H8	0.2252	0.2581	0.2104	0.069*	
C9	0.4644 (3)	0.2392 (3)	0.2932 (2)	0.0581 (6)	
H9	0.4368	0.3538	0.2687	0.070*	
C10	0.6333 (3)	0.1255 (3)	0.36490 (19)	0.0529 (6)	
C11	0.6731 (3)	−0.0434 (3)	0.4016 (2)	0.0588 (6)	
H11	0.7857	−0.1197	0.4504	0.071*	
C12	0.5455 (3)	−0.1000 (3)	0.36587 (19)	0.0548 (6)	
H12	0.5731	−0.2146	0.3902	0.066*	
C13	0.7641 (4)	0.1848 (3)	0.4034 (2)	0.0678 (7)	
C14	0.7145 (4)	−0.5000 (3)	0.0858 (3)	0.0785 (8)	
H14A	0.7910	−0.5143	0.0239	0.118*	0.50
H14B	0.6569	−0.5824	0.1090	0.118*	0.50
H14C	0.7949	−0.5136	0.1512	0.118*	0.50
H14D	0.7042	−0.5592	0.1655	0.118*	0.50
H14E	0.8383	−0.4911	0.0804	0.118*	0.50
H14F	0.7003	−0.5600	0.0382	0.118*	0.50
C15	−0.0426 (5)	0.4051 (3)	−0.3519 (2)	0.0935 (10)	
H15A	−0.1784	0.4472	−0.3465	0.140*	
H15B	−0.0203	0.3722	−0.4187	0.140*	
H15C	0.0063	0.4912	−0.3600	0.140*	

### Atomic displacement parameters (Å^2^)

**Table:**

	*U*^11^	*U*^22^	*U*^33^	*U*^12^	*U*^13^	*U*^23^
O1	0.0637 (10)	0.0628 (11)	0.0658 (11)	−0.0157 (9)	0.0118 (8)	−0.0270 (9)
O2	0.0996 (15)	0.0687 (12)	0.0564 (12)	−0.0095 (10)	−0.0079 (10)	−0.0207 (10)
O3	0.0945 (13)	0.0599 (11)	0.0487 (10)	−0.0175 (9)	0.0097 (9)	−0.0142 (9)
O4	0.0846 (13)	0.0677 (12)	0.0629 (12)	−0.0017 (10)	−0.0013 (10)	−0.0251 (10)
N1	0.0473 (10)	0.0587 (11)	0.0463 (11)	−0.0240 (9)	0.0023 (8)	−0.0206 (9)
N2	0.0470 (10)	0.0549 (11)	0.0505 (11)	−0.0196 (9)	0.0001 (9)	−0.0201 (9)
N3	0.0848 (18)	0.0871 (18)	0.114 (2)	−0.0326 (15)	−0.0303 (16)	−0.0297 (16)
C1	0.0425 (11)	0.0548 (13)	0.0514 (13)	−0.0252 (10)	0.0057 (10)	−0.0218 (11)
C2	0.0472 (12)	0.0558 (13)	0.0479 (13)	−0.0250 (11)	0.0070 (10)	−0.0216 (11)
C3	0.0470 (12)	0.0546 (13)	0.0464 (12)	−0.0260 (11)	0.0028 (10)	−0.0186 (10)
C4	0.0532 (13)	0.0561 (14)	0.0552 (15)	−0.0251 (11)	0.0009 (11)	−0.0217 (12)
C5	0.0556 (13)	0.0526 (13)	0.0524 (14)	−0.0250 (12)	0.0041 (11)	−0.0213 (11)
C6	0.0576 (14)	0.0660 (15)	0.0458 (13)	−0.0274 (12)	0.0074 (11)	−0.0210 (11)
C7	0.0491 (12)	0.0580 (13)	0.0382 (12)	−0.0194 (10)	0.0045 (9)	−0.0201 (10)
C8	0.0542 (14)	0.0533 (14)	0.0529 (14)	−0.0120 (11)	−0.0119 (11)	−0.0127 (11)
C9	0.0589 (14)	0.0514 (13)	0.0596 (15)	−0.0179 (11)	−0.0050 (12)	−0.0176 (11)
C10	0.0506 (13)	0.0596 (14)	0.0501 (13)	−0.0184 (11)	0.0016 (11)	−0.0243 (11)
C11	0.0503 (13)	0.0623 (15)	0.0535 (14)	−0.0091 (11)	−0.0097 (11)	−0.0210 (12)
C12	0.0565 (14)	0.0491 (13)	0.0527 (14)	−0.0123 (11)	−0.0022 (11)	−0.0188 (11)
C13	0.0599 (15)	0.0705 (17)	0.0711 (18)	−0.0220 (14)	−0.0092 (13)	−0.0254 (14)
C14	0.0675 (17)	0.0608 (16)	0.099 (2)	−0.0087 (14)	0.0076 (15)	−0.0363 (16)
C15	0.143 (3)	0.0669 (18)	0.0445 (15)	−0.0241 (19)	0.0060 (17)	−0.0054 (14)

### Geometric parameters (Å, °)

**Table:**

O1—C4	1.323 (3)	C7—C8	1.380 (3)
O1—C14	1.445 (3)	C8—C9	1.371 (3)
O2—C4	1.203 (3)	C8—H8	0.9300
O3—C5	1.330 (3)	C9—C10	1.386 (3)
O3—C15	1.440 (3)	C9—H9	0.9300
O4—C5	1.189 (3)	C10—C11	1.373 (3)
N1—N2	1.343 (2)	C10—C13	1.436 (3)
N1—C1	1.364 (3)	C11—C12	1.382 (3)
N1—C6	1.465 (3)	C11—H11	0.9300
N2—C3	1.345 (3)	C12—H12	0.9300
N3—C13	1.136 (3)	C14—H14A	0.9600
C1—C2	1.371 (3)	C14—H14B	0.9600
C1—C4	1.471 (3)	C14—H14C	0.9600
C2—C3	1.387 (3)	C14—H14D	0.9600
C2—H2	0.9300	C14—H14E	0.9600
C3—C5	1.468 (3)	C14—H14F	0.9600
C6—C7	1.510 (3)	C15—H15A	0.9600
C6—H6A	0.9700	C15—H15B	0.9600
C6—H6B	0.9700	C15—H15C	0.9600
C7—C12	1.376 (3)		
			
C4—O1—C14	117.1 (2)	C11—C10—C13	120.0 (2)
C5—O3—C15	115.7 (2)	C9—C10—C13	119.9 (2)
N2—N1—C1	112.02 (17)	C10—C11—C12	119.9 (2)
N2—N1—C6	117.49 (18)	C10—C11—H11	120.1
C1—N1—C6	130.25 (19)	C12—C11—H11	120.1
N1—N2—C3	104.47 (18)	C7—C12—C11	120.3 (2)
N1—C1—C2	106.61 (19)	C7—C12—H12	119.8
N1—C1—C4	123.3 (2)	C11—C12—H12	119.8
C2—C1—C4	130.0 (2)	N3—C13—C10	179.2 (3)
C1—C2—C3	105.17 (19)	O1—C14—H14A	109.5
C1—C2—H2	127.4	O1—C14—H14B	109.5
C3—C2—H2	127.4	H14A—C14—H14B	109.5
N2—C3—C2	111.72 (19)	O1—C14—H14C	109.5
N2—C3—C5	118.3 (2)	H14A—C14—H14C	109.5
C2—C3—C5	130.0 (2)	H14B—C14—H14C	109.5
O2—C4—O1	124.4 (2)	O1—C14—H14D	109.5
O2—C4—C1	125.6 (2)	H14A—C14—H14D	141.1
O1—C4—C1	110.0 (2)	H14B—C14—H14D	56.3
O4—C5—O3	123.3 (2)	H14C—C14—H14D	56.3
O4—C5—C3	126.0 (2)	O1—C14—H14E	109.5
O3—C5—C3	110.7 (2)	H14A—C14—H14E	56.3
N1—C6—C7	112.00 (17)	H14B—C14—H14E	141.1
N1—C6—H6A	109.2	H14C—C14—H14E	56.3
C7—C6—H6A	109.2	H14D—C14—H14E	109.5
N1—C6—H6B	109.2	O1—C14—H14F	109.5
C7—C6—H6B	109.2	H14A—C14—H14F	56.3
H6A—C6—H6B	107.9	H14B—C14—H14F	56.3
C12—C7—C8	119.4 (2)	H14C—C14—H14F	141.1
C12—C7—C6	120.5 (2)	H14D—C14—H14F	109.5
C8—C7—C6	120.0 (2)	H14E—C14—H14F	109.5
C9—C8—C7	120.6 (2)	O3—C15—H15A	109.5
C9—C8—H8	119.7	O3—C15—H15B	109.5
C7—C8—H8	119.7	H15A—C15—H15B	109.5
C8—C9—C10	119.6 (2)	O3—C15—H15C	109.5
C8—C9—H9	120.2	H15A—C15—H15C	109.5
C10—C9—H9	120.2	H15B—C15—H15C	109.5
C11—C10—C9	120.1 (2)		
			
C1—N1—N2—C3	1.0 (2)	C15—O3—C5—C3	−175.6 (2)
C6—N1—N2—C3	175.97 (16)	N2—C3—C5—O4	−0.4 (3)
N2—N1—C1—C2	−1.3 (2)	C2—C3—C5—O4	179.6 (2)
C6—N1—C1—C2	−175.37 (19)	N2—C3—C5—O3	179.86 (18)
N2—N1—C1—C4	−179.11 (18)	C2—C3—C5—O3	−0.1 (3)
C6—N1—C1—C4	6.8 (3)	N2—N1—C6—C7	−87.3 (2)
N1—C1—C2—C3	0.9 (2)	C1—N1—C6—C7	86.6 (3)
C4—C1—C2—C3	178.6 (2)	N1—C6—C7—C12	−113.0 (2)
N1—N2—C3—C2	−0.4 (2)	N1—C6—C7—C8	67.8 (3)
N1—N2—C3—C5	179.58 (17)	C12—C7—C8—C9	0.1 (4)
C1—C2—C3—N2	−0.3 (2)	C6—C7—C8—C9	179.2 (2)
C1—C2—C3—C5	179.7 (2)	C7—C8—C9—C10	0.1 (4)
C14—O1—C4—O2	−0.4 (3)	C8—C9—C10—C11	−0.5 (4)
C14—O1—C4—C1	179.79 (19)	C8—C9—C10—C13	−178.8 (2)
N1—C1—C4—O2	2.2 (4)	C9—C10—C11—C12	0.7 (4)
C2—C1—C4—O2	−175.2 (2)	C13—C10—C11—C12	179.0 (2)
N1—C1—C4—O1	−177.99 (18)	C8—C7—C12—C11	0.2 (3)
C2—C1—C4—O1	4.7 (3)	C6—C7—C12—C11	−179.0 (2)
C15—O3—C5—O4	4.7 (4)	C10—C11—C12—C7	−0.6 (4)

### Hydrogen-bond geometry (Å, °)

**Table:**

*D*—H···*A*	*D*—H	H···*A*	*D*···*A*	*D*—H···*A*
C6—H6A···O2	0.97	2.38	2.966 (3)	119
C14—H14A···O4^i^	0.96	2.41	3.312 (4)	156
C2—H2···Cg2^ii^	0.93	3.04	3.952 (3)	166

Symmetry codes: (i) *x*+1, *y*−1, *z*; (ii) −*x*+1, −*y*, −*z*.

### Table 2 π-π interactions in the title compound. α is the interplanar angle, DCC is the length of the vector centroid to centroid - CC), τ is the angle subtended by the plane normal to CC. Cg1 is the centroid of the ring N1\N2\C3\C2\C1; Cg2 is the centroid of the ring C7\C8\C9\C10\C11\C12.

**Table:**

Ring 1	Ring 2	α (°)	DCC (Å)	τ (°)
Cg1	Cg1^iii^	0.00	3.5074 (16)	18.75
Cg2	Cg2^iv^	0.00	3.9401 (15)	28.26

Symmetry codes: (iii) -x, -y, -z; (iv) 1-x, -y, 1-z
